# Prospective diagnostic accuracy study of plasma soluble ST2 for diagnosis of acute aortic syndromes

**DOI:** 10.1038/s41598-020-59884-6

**Published:** 2020-02-20

**Authors:** Fulvio Morello, Alice Bartalucci, Marco Bironzo, Marco Santoro, Emanuele Pivetta, Alice Ianniello, Francesca Rumbolo, Giulio Mengozzi, Enrico Lupia

**Affiliations:** 1 S.C.U. Medicina d’Urgenza, Molinette Hospital, A.O.U. Città della Salute e della Scienza, Torino, Italy; 2 Dipartimento di Scienze Mediche, Università degli Studi di Torino, Torino, Italy; 3 S.C. Biochimica Clinica, A.O.U. Città della Salute e della Scienza, Torino, Italy

**Keywords:** Aortic diseases, Diagnostic markers

## Abstract

Acute aortic syndromes (AASs) are difficult to diagnose emergencies. Plasma soluble ST2 (sST2), a prognostic biomarker for heart failure, has been proposed as a diagnostic biomarker of AASs outperforming D-dimer, the current diagnostic standard. We performed a prospective diagnostic accuracy study of sST2 for AASs in the Emergency Department (ED). In 2017–2018, patients were enrolled if they had ≥1 red-flag symptoms (chest/abdominal/back pain, syncope, perfusion deficit) and a clinical suspicion of AAS. sST2 was detected with the Presage® assay. Adjudication was based on computed tomography angiography (CTA) or on diagnostic outcome inclusive of 30-day follow-up. 297 patients were enrolled, including 88 with AASs. The median age was 67 years. In 162 patients with CTA, the median sST2 level was 41.7 ng/mL (IQR 29.4–103.2) in AASs and 34.6 ng/mL (IQR 21.4–51.5) in alternative diagnoses (*P* = 0.005). In ROC analysis, the AUC of sST2 was 0.63, as compared to 0.82 of D-dimer (*P* < 0.001). Sensitivity and specificity values of sST2 associated with different cutoffs were: 95.5% and 10.8% (≥12 ng/mL), 84.1% and 29.7% (≥23.7 ng/mL), 35.2% and 85.1% (≥66.5 ng/mL). Results were similar in the full cohort. In conclusion, in patients from a European ED, plasma sST2 provided modest accuracy for diagnosis of AASs.

## Introduction

Acute aortic syndromes (AASs), which include acute aortic dissection (AAD), intramural aortic hematoma (IMH), penetrating aortic ulcer (PAU) and spontaneous aortic rupture (SAR), are severe cardiovascular emergencies affecting 5–10 per 100.000 individuals a year^1^. A conclusive diagnosis of AASs requires advanced aortic imaging, typically computed tomography angiography (CTA), which uses ionizing radiations and may cause contrast-induced nephropathy and anaphylaxis. However, selection of the right patient to image with CTA is cumbersome, because AASs present with unspecific symptoms such as truncal pain, syncope, neurological deficits and hindlimb ischemia. These are leading causes of Emergency Department (ED) visits worldwide, and most patients with these symptoms are affected by more common conditions, such as acute coronary syndromes, gastrointestinal diseases and muscle-skeletal pain. Hence, the misdiagnosis rate of AASs is high, affecting clinical outcomes^2–4^. Increasing use of CTA in EDs has led to a high number of CTA exams requested for suspected AAS turning out negative, but has not substantially changed misdiagnosis rates, indicating that pre-test patient selection remains a key node^5–7^. Development of reliable diagnostic biomarkers discriminating AASs could positively impact on their diagnostic workup, as for other cardiovascular emergencies such as acute coronary syndromes and pulmonary embolism^8,9^.

So far, potential biomarkers of AASs have been searched for within aortic layers (*e.g*. smooth muscle myosin, soluble elastin fragments, calponin, CD40 ligand), within inflammation/remodeling pathways (*e.g*. matrix metalloproteinases) and within coagulation processes mobilized by blood exposure to non-endothelial surfaces (*e.g*. platelets, D-dimer)^10^. Currently, D-dimer is the only biomarker clinically applicable to the workup of AASs^11,12^. Owing to high sensitivity, D-dimer can be used for diagnostic rule-out of AASs, if the pre-test probability is sufficiently low^13^. However, D-dimer can be increased by several conditions such as inflammation, infection, cancer, trauma and age, leading to low diagnostic specificity. On these grounds, the quest for additional diagnostic biomarkers for AASs remains open and vibrant.

ST2 (suppression of tumorigenicity 2, encoded by interleukin 1 receptor-like 1 or IL1RL-1) is a member of the interleukin-1 receptor family, regulating immune-responses^14^. Soluble ST2 (sST2), a truncated form of transmembrane ST2, is secreted into the circulation and functions as a decoy receptor for interleukin-33. Evidence has accumulated that sST2 is up-regulated in cardiac cells upon myocardial strain and several studies have established plasma sST2 as a biomarker for clinical risk prediction in patients affected by heart failure^15–19^. The role of sST2 within vascular tissues and in the context of aortic disease is largely unknown^20^. However, Wang *et al*. recently reported that plasma sST2, measured with a research-grade assay, showed striking diagnostic accuracy for AASs and outperformed D-dimer, in a Chinese patient cohort^21^. Since plasma sST2 levels can be measured with a clinical-grade assay already approved for patient use, we sought to further evaluate it as a diagnostic biomarker for AASs in an independent prospective cohort of acute patients from a European ED.

## Results

### Study population

In the study period, 297 patients with any pre-defined red-flag symptom (chest/abdominal/back pain, syncope, perfusion deficit) and a clinical suspicion of an AAS, were enrolled. Presenting symptoms were the following: anterior chest pain (189, 63.6%), posterior chest pain (114, 38.4%), abdominal pain (66, 22.2%), back pain (25, 8.4%), syncope (34, 11.4%), perfusion deficit (20, 6.7%). The demographic and clinical characteristics of study patients are reported in Table 1. The prevalence of risk factors for AASs and the corresponding aortic dissection detection risk score are presented in Table 2. 243 patients (81.8%) were classified at low pre-test clinical probability of AAS, according to ESC guidelines^13^.

**Table 1:** Demographic and clinical characteristics of prospectively enrolled patients.

Variable	Total patients (n = 297)	AASs (n = 88)	AltDs (n = 209)	*P*-value
Female gender	94 (31.6%)	26 (29.5%)	68 (32.5%)	0.61
Age [y]	67 (55–78)	72 (59.5–80)	65 (53–77)	0.04
Hypertension	176 (59.3%)	62 (70.5%)	114 (54.5%)	0.011
Diabetes	27 (9.1%)	7 (8%)	20 (9.6%)	0.66
Dyslipidemia	18 (6.1%)	3 (3.4%)	15 (7.2%)	0.21
Smoking	87 (29.3%)	26 (29.5%)	61 (29.2%)	0.95
Drug use	3 (1%)	2 (2.3%)	1 (0.5%)	0.16
Coronary art. dis.	37 (12.5%)	6 (6.8%)	31 (14.8%)	0.06
Active cancer	2 (0.7%)	0 (0%)	2 (1%)	0.36
Periph. art. dis.	1 (0.3%)	0 (0%)	1 (0.5%)	0.52
Abdominal aortic an.	14 (4.7%)	5 (5.7%)	9 (4.3%)	0.61
Previous AAS	10 (3.4%)	4 (4.5%)	6 (2.9%)	0.47
Systolic BP [mmHg]	140 (120–160)	140 (110–170)	140 (125–160)	0.48
Diastolic BP [mmHg]	80 (70–90)	80 (60–95)	80 (75–90)	0.04
Heart rate [bpm]	75 (68–87)	72 (64–83)	75 (70–89)	0.15
Time from onset [h]	4 (2–11)	3 (2–6)	5 (2–14)	0.02
WBC count [*10^3^/μL]^A^	9.07 (7.22–11.71)	11.66 (9.1–14.35)	8.14 (6.78–10.45)	<0.001
Creatinine [mg/dL]^B^	0.97 (0.82–1.16)	1.06 (0.86–1.21)	0.93 (0.81–1.12)	0.004
Troponin T [ng/mL]^C^	11 (7–23)	20 (10–41)	10 (5–18)	<0.001
D-dimer [ng/mL]^D^	780 (331.5–3466.5)	6301 (1813–28646)	471 (265.5–1002.5)	<0.001

^A^n = 294; ^B^n = 293; ^C^n = 262; ^D^n = 211. Categorical variables are presented as n (%) and continuous variables as median (25^th^-75^th^ percentile). AASs = acute aortic syndrome; AltDs: alternative diagnoses; an. = aneurysm; art. = artery; BP = blood pressure; dis. = disease; sy. = syndrome; thor. = thoracic.

**Table 2:** Aortic dissection detection risk factors and associated risk score of study patients.

Variable	Total patients (n = 297)	AASs (n = 88)	AltDs (n = 209)	*P*-value
**History**				
Marfan/conn. tissue dis.	1 (0.3%)	0 (0%)	1 (0.5%)	0.52
Family history of AAS	7 (2.4%)	2 (2.3%)	5 (2.4%)	0.95
Aortic valve disease	21 (7.1%)	7 (8%)	14 (6.7%)	0.7
Recent aortic manipulation	3 (1%)	1 (1.1%)	2 (1%)	0.89
Thoracic aortic aneurysm	40 (13.5%)	21 (23.9%)	19 (9.1%)	0.001
**Pain**				
Sudden pain	146 (49.2%)	55 (62.5%)	91 (43.5%)	0.001
Severe pain	73 (24.6%)	28 (31.8%)	45 (21.5%)	0.003
Ripping/tearing pain	32 (10.8%)	20 (22.7%)	12 (5.7%)	0.06
**Physical findings**				
Pulse deficit	23 (7.7%)	16 (18.2%)	7 (3.3%)	<0.001
Neurologic deficit	13 (4.4%)	7 (8%)	6 (2.9%)	0.05
New diastolic murmur	1 (0.3%)	1 (1.1%)	0 (0%)	0.12
Hypotension	18 (6.1%)	15 (17%)	3 (1.4%)	<0.001
**ADD risk score**				
ADD risk score = 0	88 (29.7%)	11 (12.5%)	77 (36.8%)	<0.001
ADD risk score = 1	155 (52.2%)	45 (51.1%)	110 (52.6%)	0.81
ADD risk score = 2–3	54 (18.2%)	32 (36.4%)	22 (10.5%)	<0.001

AASs: acute aortic syndromes; ADD: aortic dissection detection; AltDs: alternative diagnoses; conn.: connective; dis.: disease.

### Diagnostic work-up and case adjudication

**Figure 1:**
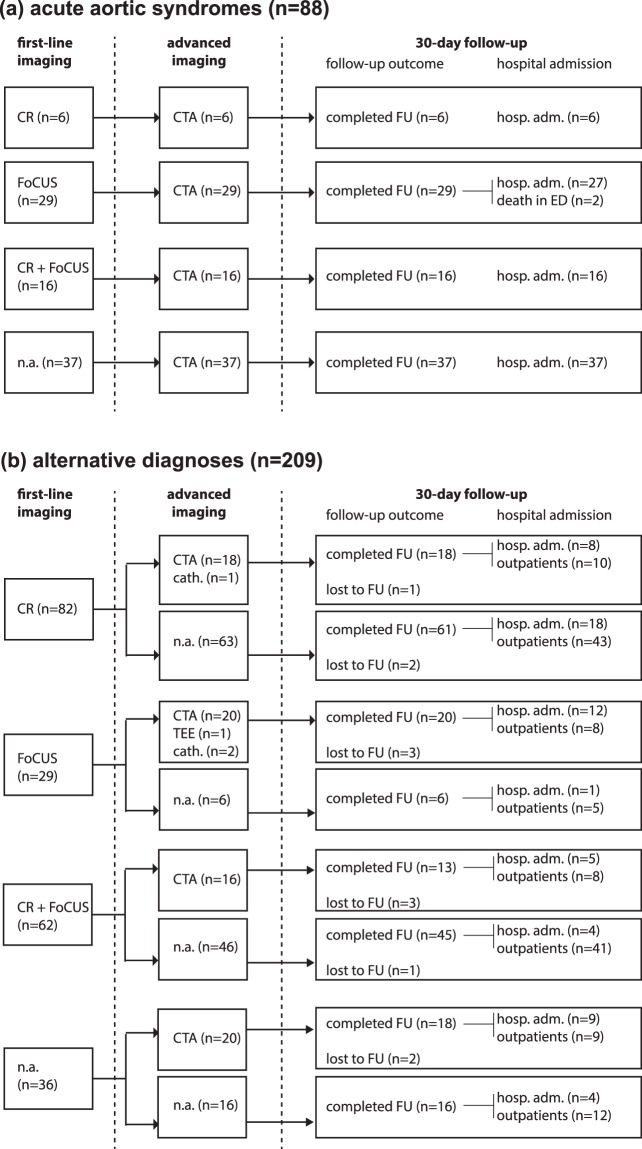
Data used for diagnostic outcome adjudication. Cath: coronary angiography (cardiac catheterization laboratory); CR: chest X-ray; CTA: contrast-enhanced computed tomography angiography of the chest and abdomen; FoCUS: focused cardiac ultrasound; FU: follow-up; hosp. adm.: hospital admission; n.a.: not available; TEE: transesophageal echocardiography.

The diagnostic workup conducted during the index ED visit included chest X-ray in 166 (55.9%) patients, focused transthoracic echocardiography in 136 (45.8%), CTA in 162 (54.5%), transesophageal echocardiography (TEE) in 1 (0.3%) and coronary angiography in 3 (1%). 30-day follow-up data was obtained for 287 (96.6%) patients. 147 (49.5%) patients were admitted to hospital. Amongst 10 patients lacking follow-up data, 7 were subjected to advanced aortic imaging in the ED excluding an AAS. Diagnostic data allowing final case adjudication are detailed in Fig. 1.

An AAS was adjudicated in 88 (29.6%) patients, all subjected to CTA. The AAS types were: 46 (15.5%) Stanford type A AADs, 20 (6.7%) Stanford type B AADs, 18 (6.1%) IMHs, 3 (1%) SARs and 1 (0.3%) PAU. In 209 (70.4%) patients, an AAS was excluded and the following alternative diagnoses (AltDs) were adjudicated: skeletal-muscle pain (45, 15.2%), gastrointestinal disease (28, 9.4%), acute coronary syndrome (18, 6.1%), non AAS-related syncope (16, 5.4%), other aortic disease (15, 5.1%), pericarditis (12, 4%), pleurisy/pneumonia (9, 3%), stroke (1, 0.3%), pulmonary embolism (1, 0.3%) and other conditions (64, 21.5%). The demographic and clinical characteristics of study patients classified by diagnostic outcome are reported in Table 1. Patients affected by AASs had significantly higher levels of white blood cells (WBCs), creatinine, troponin and D-dimer.

### CTA-based adjudication

**Figure 2:**
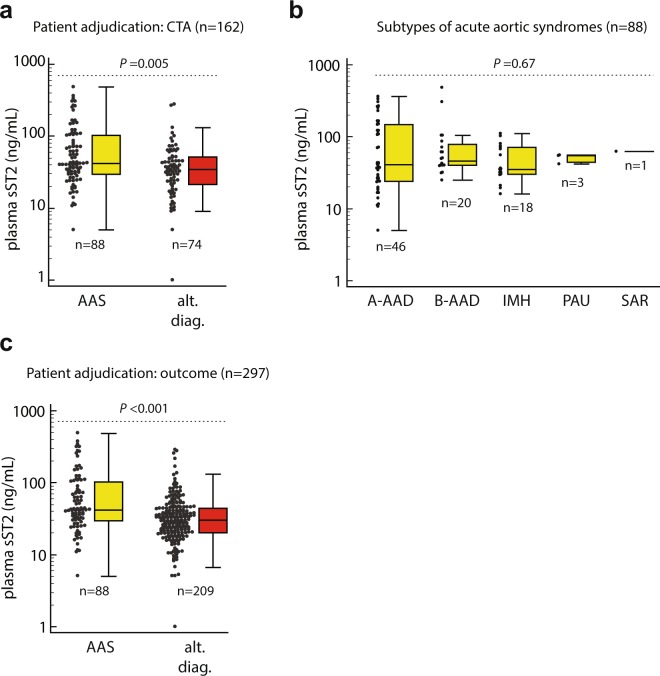
Dot-plot and box-whisker representation of plasma sST2 levels in study patients, classified by: **(a)** dichotomic final diagnosis (adjudication based on CTA), **(b)** subtype of acute aortic syndrome, **(c)** dichotomic final diagnosis (adjudication based on diagnostic outcome). A-AAD: type A acute aortic dissection; AAS: acute aortic syndrome; ACS: acute coronary syndrome; alt. diag.: alternative diagnosis; B-AAD: type B AAD; IMH: intramural aortic hematoma; PAU: penetrating aortic ulcer; SAR: spontaneous aortic rupture.

We first analyzed the diagnostic characteristics of plasma sST2 within patients subjected to CTA (n = 162, 88 with AASs), which represents the imaging gold standard to rule-in/out AASs. The demographic and clinical characteristics of patients with AltDs subjected to CTA (n = 74) are reported in Supplementary Table 1. The median plasma sST2 level was 41.7 ng/mL (IQR 29.4–103.2) in patients with AASs and 34.6 ng/mL (IQR 21.4–51.5) in patients with AltDs (*P* = 0.005, Fig. 2a). Median sST2 levels were 40.8 ng/mL (IQR 24–146.8) in type A AADs, 45.7 ng/mL (IQR 40–79.2) in type B AADs, 35.2 ng/mL in IMHs (IQR 30.1–71.5), 54.2 ng/mL (IQR 44.7–55.3) in SARs and 61.9 ng/mL in PAUs (*P* = 0.67; Fig. 2b). The levels of sST2 in different types of AltDs did not significantly differ (*P* = 0.34, Supplementary Fig. 1a).

**Figure 3:**
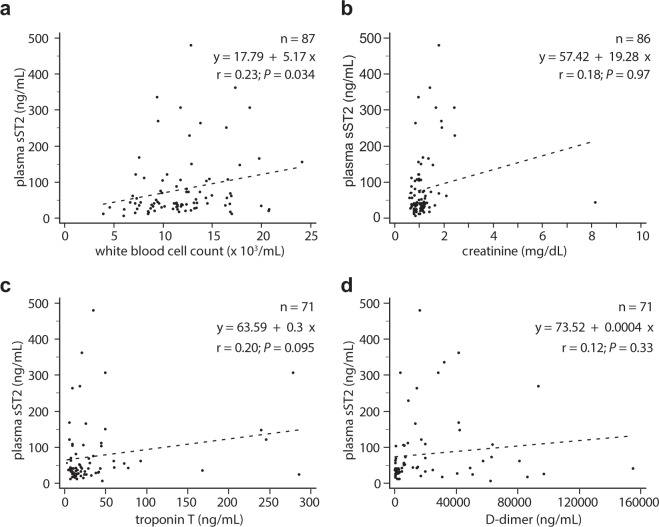
Scatter plots evaluating the correlation and linear regression between plasma sST2 and **(a)** white blood cell count, **(b)** creatinine, **(c)** troponin T and **(d)** D-dimer, in patients with acute aortic syndromes. Linear regression analysis data are presented as inset.

sST2 levels showed linear correlation with WBC count (r = 0.3, *P* < 0.001), creatinine (r = 0.19, *P* = 0.018) and D-dimer (r = 0.21, *P* = 0.018), but not with troponin T (r = 0.14, *P* = 0.1), and non-linear correlation with all variables (WBC count, ρ_s_ = 0.26, *P* < 0001.; creatinine, ρ_s_ = 0.19, *P* = 0.014; D-dimer, ρ_s_ = 0.36, *P* < 0.001; troponin, ρ_s_ = 0.25, *P* = 0.04). In patients with AASs, sST2 levels showed linear correlation with WBC count (Fig. 3), and non-linear correlation with WBC count (ρ_s_ = 0.26, *P* = 0.014), creatinine (ρ_s_ = 0.27, *P* = 0.014) and D-dimer (ρ_s_ = 0.28, *P* = 0.017), but not with troponin T (ρ_s_ = 0.17, *P* = 0.17). In patients with AltDs, sST2 levels showed linear correlation with WBC count (r = 0.30; *P* = 0.009) and D-dimer (r = 0.26; *P* = 0.04), but not with creatinine (r = 0.06; *P* = 0.64) and troponin (r = 0.2; *P* = 0.1); non-linear correlation was found with troponin (ρ_s_ = 0.29, *P* = 0.017), but not with WBC count (ρ_s_ = 0.09, *P* = 0.46), creatinine (ρ_s_ = 0.08, *P* = 0.52) and D-dimer (ρ_s_ = 0.24, *P* = 0.06).

**Figure 4:**
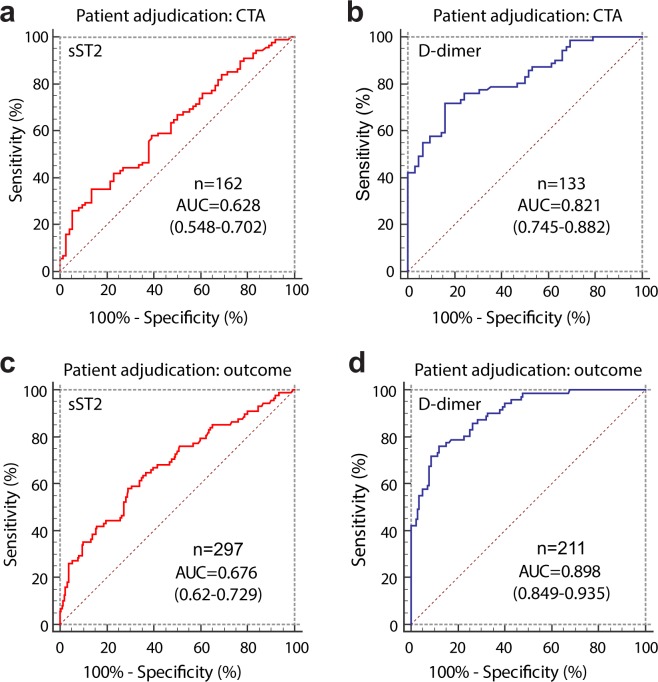
Receiver operating characteristic (ROC) curves of plasma sST2 and D-dimer for diagnosis of acute aortic syndromes in **(a,b)** patients subjected to CTA and **(c,d)** in all study patients classified according to diagnostic outcome. AUC values are reported with their 95%CI in brackets.

The receiver operating characteristic (ROC) curves of sST2 and D-dimer for diagnosis of AASs are shown in Fig. 4a,b. The area under the curve (AUC) values of sST2 and D-dimer significantly differed (*P* < 0.001). In patients sampled within 24 hours from symptom onset, the AUC of sST2 (n = 118) was 0.591 (95%CI 0.497–0.681), while the AUC of D-dimer (n = 97) was 0.842 (95%CI 0.753–0.908; *P* < 0.001 *vs* sST2). In patients at low clinical probability of AAS (as defined by ADD-RS ≤ 1), the AUC of sST2 (n = 120) was 0.661 (95%CI 0.569–0.745), while the AUC of D-dimer (n = 98) was 0.768 (95%CI 0.672–0.848; *P* = 0.12 *vs* sST2).

Using a conventional cutoff of 500 ng/mL, D-dimer had a sensitivity of 95.8% (95%CI 88.1–99.1%), a specificity of 30.7% (95%CI 19.6–43.7%), a LR+ of 1.38 (95%CI 1.2–1.6) and a LR− of 0.14 (95%CI 0.04–0.4) for AASs. For sST2, the cutoff maximizing the sensitivity and specificity was 66.5 ng/mL. Table 3 reports the diagnostic test characteristics of sST2 for AASs at different selected cutoffs.

**Table 3:** Diagnostic test characteristics of sST2 for diagnosis of acute aortic syndromes.

Variable	Case adjudication	sST2 ≥ 12 ng/mL^A^	sST2 ≥ 23.7 ng/mL^B^	sST2 ≥ 66.5 ng/mL^C^
Sensitivity (%)	CTA (n = 162) and diagnostic outcome (n = 297)	95.5% (95%CI 88.8–98.7%)	84.1% (95%CI 74.8–91%)	35.2% (95%CI 25.3–46.1%)
Specificity (%)	CTA (n = 162)	10.8% (95%CI 4.8–20.2%)	29.7% (95%CI 19.7–41.5%)	85.1% (95%CI 75–92.3%)
	diagnostic outcome (n = 297)	8.6% (95%CI 5.2–13.3%)	35.4% (95%CI 28.9–42.3%)	89.5% (95%CI 84.5–93.3%)
LR+	CTA (n = 162)	1.07 (95%CI 1–1.2)	1.20 (95%CI 1–1.4)	2.37 (95%CI 1.3–4.4)
	diagnostic outcome (n = 297)	1.04 (95%CI 1.0–1.1)	1.3 (95%CI 1.1–1.5)	3.35 (95%CI 2.1–5.4)
LR−	CTA (n = 162)	0.42 (95%CI 0.1–1.3)	0.54 (95%CI 0.3–1)	0.76 (95%CI 0.6–0.9)
	diagnostic outcome (n = 297)	0.53 (95%CI 0.2–1.5)	0.45 (95%CI 0.3–0.8)	0.72 (95%CI 0.6–0.9)

^A^Cutoff providing similar sensitivity to D-dimer (cutoff 500 ng/mL) in the CTA-based analysis; ^B^cutoff providing similar specificity to D-dimer (cutoff 500 ng/mL) in the CTA-based analysis; ^C^cutoff maximizing sensitivity and specificity (Youden’s method) in the CTA-based analysis. LR: likelihood ratio.

### Diagnostic outcome-based adjudication

We next evaluated the diagnostic test characteristics of plasma sST2 in the whole study cohort *per* prospective enrolment and diagnostic outcome adjudication (n = 297, Tables 1 and 2). The median plasma sST2 level was 41.7 ng/mL (IQR 29.4–103.2) in AASs and 30.1 ng/mL (IQR 20–43.8) in AltDs (*P* < 0.001; Fig. 2c). The levels of sST2 in different types of AltDs did not significantly differ (*P* = 0.44; Supplementary Fig. 1b).

In patients with AltDs, sST2 showed linear correlation with WBC count (r = 0.24, *P* < 0.001), creatinine (r = 0.22, *P* = 0.001), D-dimer (r = 0.42, *P* < 0.001) and troponin (r = 0.17, *P* = 0.02); non-linear correlation was found with creatinine (r = 0.16, *P* = 0.025), D-dimer (r = 0.25, *P* = 0.03) and troponin (r = 0.19, *P* = 0.09), but not with WBC count (r = 0.05, *P* = 0.43). In patients with an AltD of acute coronary syndrome, plasma sST2 levels showed non-linear correlation with troponin T (ρ_s_ = 0.63, *P* = 0.01), but not with WBC count (ρ_s_ = 0.17, *P* = 0.51), creatinine (ρ_s_ = 0.32, *P* = 0.22) and D-dimer (ρ_s_ = 0.04, *P* = 0.89).

The ROC curves of sST2 and D-dimer for diagnosis of AASs in the full study cohort are shown in Fig. 4c,d. The AUC values of sST2 and D-dimer significantly differed (*P* < 0.001). In patients sampled within 24 hours from symptom onset, the AUC of sST2 (n = 223) was 0.675 (95%CI 0.61–0.736), while the AUC of D-dimer (n = 160) was 0.917 (95%CI 0.863–0.955; *P* < 0.001 *vs* sST2). In patients at low clinical probability of AAS, the AUC of sST2 (n = 243) was 0.717 (95%CI 0.655–0.772), while the AUC of D-dimer (n = 168) was 0.867 (95%CI 0.806–0.914; *P* = 0.002 *vs* sST2).

Using a cutoff of 500 ng/mL, D-dimer had a sensitivity of 95.8% (95%CI 88.1–99.1%), a specificity of 53.6% (95%CI 45–62%), a LR + of 2.06 (95%CI 1.7–2.5) and a LR− of 0.079 (95%CI 0.03–0.2) for AASs. The diagnostic test characteristics of sST2 for AASs in the full study cohort are reported in Table 3. The cutoff maximizing the sensitivity and specificity of sST2 for AASs in the full cohort was 39.8 ng/mL. The diagnostic test characteristics associated with sST2 ≥ 39.8 ng/mL were: sensitivity 58% (95%CI 47–68.4%), specificity 70.8% (95%CI 64.1–76.9%), positive likelihood ratio (LR+) 1.99 (95%CI 1.5–2.6), negative likelihood ratio (LR−) 0.59 (95%CI 0.5–0.8).

## Discussion

In this single-center prospective study performed in a European ED, median plasma levels of sST2 measured with a clinical-grade assay were higher in patients with AASs than in patients with AltD. However, there was high inter-patient variability and the overall performance of sST2 for diagnosis AASs was modest. Furthermore, sST2 proved inferior to D-dimer, a well-established biomarker of AASs already applied in clinical practice.

The present study post-dated the study by Wang *et al*. reporting high sensitivity and specificity of plasma sST2 for AASs and indicating potential superiority of sST2 to D-dimer^21^. Several issues may cause the discrepancy between current and previous data. First, the present study involved a larger case-mix and an elder population, both typical of western country hospitals. Indeed, a recent study has reported significant variation in sST2 levels related to age, with 50 years as a critical cutoff^22^. Major differences in age between the present and the previous study may account for the discrepancy. Second, male gender has also been associated with higher sST2 levels. However, since gender distribution was similar between the two studies, this should not be regarded as a major confounder. Third, ethnic differences in sST2 levels between Asian and European populations may exist. Indeed, the lowest diagnostic accuracy estimates for D-dimer have been obtained in Asian studies^23^. Finally, the present study used a different assay for detection of sST2, *i.e*. the only one approved for patient use^24^. In Wang *et al*., the results of the research-grade R&D assay and the clinical-grade Presage assay strongly correlated. In the present study, we also found a significant albeit looser correlation between assays. Technical differences between sST2 assays have already been documented^25^. However, additional ethinc specific differences between assays are also conceivable.

Together with previous evidence, current findings indicate that AASs may cause release of sST2 into the bloodstream, but the source and kinetics of this process remain largely unknown. In the present study, the levels of sST2 did not correlate with those of troponin T in AASs, indicating a loose relationship, if any, between plasma sST2 levels and myocardial damage. Instead, the levels of plasma sST2 and troponin T were correlated in patients with AltDs (including subgroup with acute coronary syndromes). In AASs, the levels of plasma sST2 correlated with WBC count and D-dimer, rather suggesting a pathophysiological link with inflammatory pathways engaged by acute aortic lesions^8,26,27^. Also in the study by Wang *et al*., plasma sST2 positively correlated with D-dimer concentrations and not with those of brain natriuretic peptide.

The present study has limitations. First, clinical suspicion of AASs, representing a key patient selection criterion, falls short of a universal definition. Since the study was performed in a tertiary ED specialized in cardiovascular emergencies, variation of physician’s gestalt and local practice might limit result generalizability. Second, due to ethical and clinical considerations (exposure to radiation and contrast medium), CTA could not be performed as uniform gold standard. Treating physicians decided on CTA based on patient-level clinical reasoning irrespective of study participation, in compliance with an observational and non-interventional study protocol. A follow-up approach was used for pragmatic case adjudication based on 30-day outcomes. This method is routinely applied in diagnostic studies of cardiovascular emergencies in the ED such as pulmonary embolism and acute coronary syndromes and has been used in a previous multicenter study of AASs^12,28–32^. As compared to our previous work, the timeline of the follow-up was extended from 14 to 30 days, in order to fully cover both the acute and subacute phases of AASs. Very few patients with clinically mild forms of AASs (*e.g*. short uncomplicated IMHs or PAUs) might have been misdiagnosed. However, they could not substantially affect the study results. Accordingly, the results obtained in the CTA subgroup and in the full cohort did not differ.

In conclusion, in a European cohort of ED patients, plasma sST2 showed poor diagnostic accuracy for AASs. D-dimer, instead, confirmed its established diagnostic performance characterized by high sensitivity and moderate specificity. Results substantially differ from those previously obtained in a larger and much younger cohort of Chinese patients. Further studies are needed to evaluate sST2 as a potential biomarker of aortic diseases in younger patients of non-Asian ethnicity.

## Materials and Methods

### Study design

We conducted a single-center prospective diagnostic accuracy study, in a large urban ED. The methods were carried out in accordance with the relevant guidelines and regulations. The local Human Ethics Committee (Comitato Etico Interaziendale A.O.U. Città della Salute e della Scienza di Torino) approved the study and written informed consent was obtained from participants or their authorized representatives. The study protocol conformed to the ethical guidelines (Declaration of Helsinki, 1975).

### Selection of participants

From January 2017 to October 2018, outpatients aged ≥18 years were enrolled: (1) if they presented ≥1 red-flag symptom(s) of AAS (chest/abdominal/back pain, syncope, perfusion deficit) dating ≤14 days, and (2) if an AAS was considered in differential diagnosis by the attending physician. To allow immediate handling and storage of plasma samples in the laboratory, enrolment was limited to daytime, from 8 a.m. to 8 p.m., 7 days/week.

### Laboratory analyses

During the ED visit, patients underwent venipuncture for the routine blood tests deemed necessary by the attending physician. Blood samples were immediately sent to the local laboratory for automatic processing. D-dimer levels were measured urgently by the laboratory on trisodium citrate-plasma samples, using the STA-Liatest D-Di assay (Stago, Asnières sur Seine, France). This is a rapid, automated, quantitative immune-turbidimetric quantitative assay. The results of the D-dimer assay were available live to the attending physicians, for clinical decisions. For sST2 measurement, for each patient an EDTA-plasma sample aliquot was rapidly obtained in the laboratory and stored at −80 °C until further processing. The levels of sST2 were measured subsequently on thawed plasma batches and were not available to attending physicians for clinical use.

### sST2 assay

The concentrations of plasma sST2 were measured using the Critical Diagnostics Presage® ST2, a quantitative sandwich type ELISA (enzyme-linked immunosorbent assay) which uses monoclonal antibodies. Human endogenous ST2 demonstrated stability in all the following conditions: storage at 20 °C for 48 h, at 4 °C for 7 days, at −20 °C and −80 °C for 18 months. The assay precision assessment was performed according to the CLSI (Clinical and Laboratory Standards Institute) EP5-A standards. Intra-series coefficient of variation (CVa) and total CVa were 6.5% and 9.1%, respectively, at an average concentration of 16.9 ng/mL, 3.4% and 5.5% at a mean concentration of 33.1 ng/mL, 3.8% and 6.3% at an average concentration of 68.7 ng/mL, 2.4% and 4.8% at an average concentration of 159.1 ng/mL. The limit of quantification is 2.4 ng/mL and the dynamic range of measurement is from 2.4 ng/mL up to 200 ng/mL. There are no significant interferences with total proteins, triglycerides, hemoglobin, cholesterol, bilirubin.

In a subset of 86 patients (24 AAS, 62 AltD) with sufficient plasma aliquots, sST2 levels were assayed also with the human ST2/IL-1 R4s assay by R&D Systems (DY523B-05; Minneapolis, MN, USA), according to the manufacturer’s protocol. Passing and Bablok regression analyses were used for method comparison. The Cusum test showed non-significant deviation from linearity (p = 0.88). Non-parametric correlation analysis revealed a Spearman’s coefficient of rank correlation (ρ_s_) of 0.29 (95%CI, 0.11–0.45; *P* = 0.003) between results obtained with the Presage and the R&D assay.

### Case adjudication

All diagnostic and clinical decisions were performed at the discretion of the treating providers, independent of study participation. For each patient, a dichotomic case adjudication (AAS *vs* alternative diagnosis, AltD) was made by two independent reviewers expert in Emergency Medicine and acute aortic syndromes, blinded to sST2 and D-dimer levels, who evaluated all available clinical data (ED/hospital/surgical charts, imaging data, blood test results) obtained within 30 days after the index ED visit, including the results of a 30-day clinical or structured telephone follow-up contact performed by a research staff. In case of discordance, cases were adjudicated after discussion. Patients were instructed to return to the ED in case of new, worsening or recurrent symptoms.

AAS was defined by evidence of an AAS on CTA, TEE, magnetic resonance angiography, surgery or autopsy. If advanced imaging data, surgical or autopsy data was not available, an AAS was considered absent: (1) in patients admitted to hospital, if an alternative diagnosis was available, (2) in patients dismissed from the ED, if they had an uncomplicated clinical course or if an alternative diagnosis was made during the 30-day follow-up period.

### Outcome measures and sample size

Primary outcomes were the diagnostic test characteristics of sST2 for AASs. To calculate sample size, we hypothesized to test an AUC difference of 10% between sST2 and D-dimer, for diagnosis of AASs. Based on metanalysis of available studies, we assumed for D-dimer an AUC of 95%^23^. Using an alpha of 0.05 (one sided) and a power of 0.8, we needed to include about 250 patients to reject the null hypothesis.

### Data analysis

Continuous variables were expressed as median and IQR (interquartile range, indicated as 25^th^ to 75^th^ percentile values). Dichotomous data were expressed as proportions (in % value) with their 95% confident interval (CI), computed using Wilson’s method. The diagnostic performance of the biomarkers was evaluated with ROC analysis. AUCs were calculated and compared *per* Hanley & McNeil. For specified cutoffs, sensitivity, specificity and positive/negative likelihood ratios (LR+/LR−) were computed with their 95%CI. The tests used for statistical comparison were Pearson’s χ^2^ test (dichotomous data), Mann-Whitney U-test for independent samples (continuous data, two-groups) and Kruskal-Wallis test for independent samples (continuous data, multiple groups). *P*-values were two-sided and *P* < 0.05 was considered significant. The analysis was performed with SPSS statistical package (ver. 25.0, SPSS Inc., Chicago, Illinois) and MedCalc software (ver 18.11.3).

## Supplementary information

Supplementary data file.

## Data Availability

The datasets generated during and analyzed during the current study are available from the corresponding author on reasonable request.
