# Supplementary material for: Prospective diagnostic accuracy study of plasma soluble ST2 for diagnosis of acute aortic syndromes
Source: Sci Rep. 2020 Feb 20;10:3103. doi: 10.1038/s41598-020-59884-6 (PMC7033105; doi:10.1038/s41598-020-59884-6)
Supplement: Supplementary file 1 — Supplementary data file. [file 41598_2020_59884_MOESM1_ESM.pdf]

Prospective diagnostic accuracy study of plasma soluble ST2 for diagnosis of acute aortic syndromes

Fulvio Morello M.D. Ph.D.[1,2], Alice Bartalucci M.D.[1], Marco Bironzo M.D.[1], Marco Santoro M.S.[1], Emanuele Pivetta M.D. Ph.D.[1], Alice Ianniello B.D.[3], Francesca Rumbolo B.D.[3], Giulio Mengozzi M.D. Ph.D.[3], Enrico Lupia M.D. Ph.D.[1,2]

1. S.C.U. Medicina d'Urgenza, Molinette Hospital, A.O.U. Città della Salute e della Scienza, Torino, Italy. 2. Dipartimento di Scienze Mediche, Università degli Studi di Torino, Torino, Italy. 3. S.C. Biochimica Clinica, A.O.U. Città della Salute e della Scienza, Torino, Italy.

SUPPLEMENTARY MATERIAL

Supplementary figure 1.

a

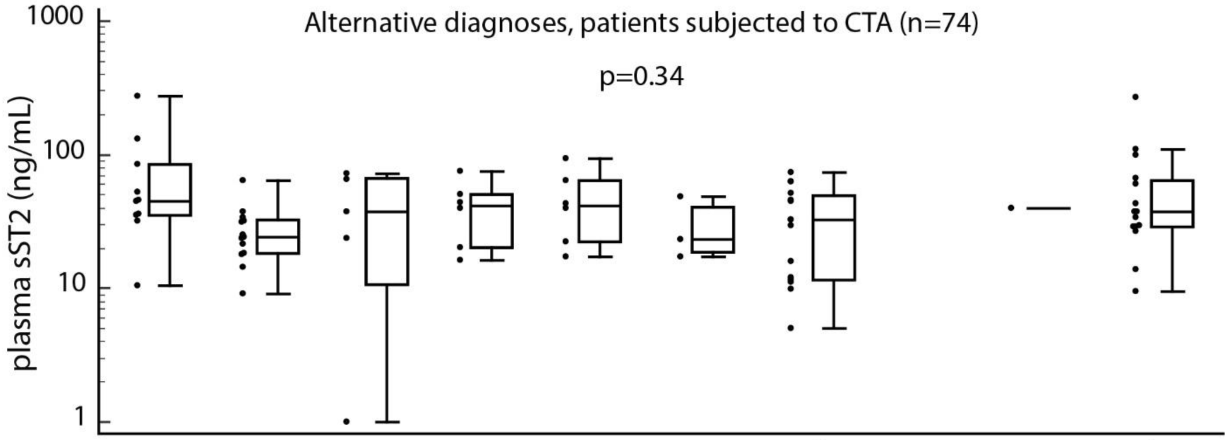

	GI dis.	MS pain	Peric	Pneu	ACS	Sync	Other aortic	PE	Other
n	10	13	5	6	6	3	15	1	15
Median	45	24,2	37,4	41,5	41,2	23,3	32,3	39,9	37,2
IQR	35,1-84,3	18,2-32,3	18,1-67,1	20,1-50,4	22,1-64,7	18,8-42,4	11,6-50		28,8-64,8

b

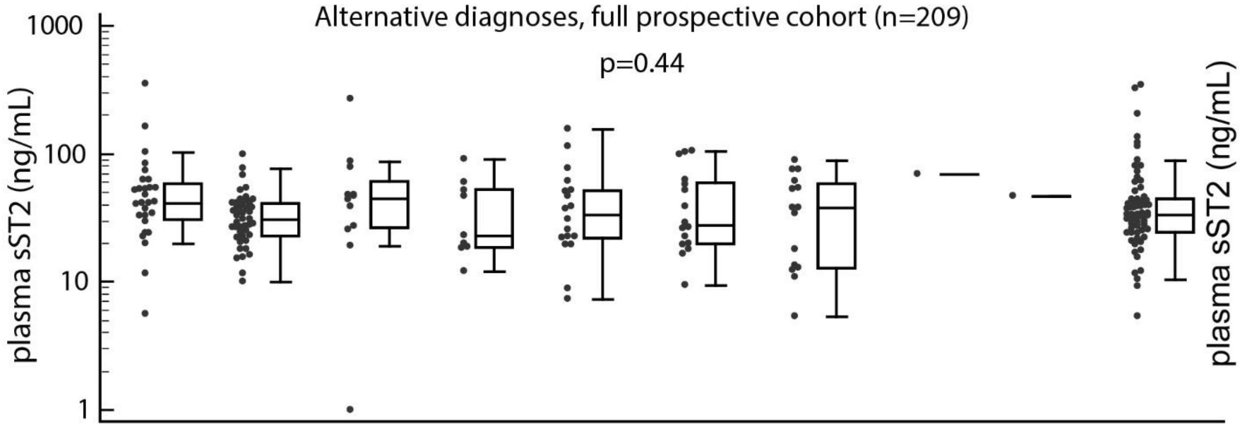

	GI dis.	MS pain	Peric	Pneu	ACS	Sync	Other aortic	Stroke	PE	Other
n	28	45	12	9	18	16	15	1	1	64
Median	35,6	26,5	38,6	20,1	29,2	24,2	32,3	57,9	39,9	28,9
IQR	26,9-49,3	20,1-35,2	23,1-53	16,6-45,2	19,5-43,7	17,5-50,4	11,6-50			21,5-38,2

Figure legend. Dot-plot and box-whisker representation of plasma sST2 levels in study patients with alternative diagnoses. **(a)** Patients subjected to CTA. **(b)** Patient adjudication based on diagnostic outcome (full cohort). ACS: acute coronary syndrome; GI dis: gastro-intestinal disease; MS: muscle-skeletal; Other aortic: other aortic diseases; PE: pulmonary embolism; Peric: pericarditis; Pneu: pleurisy/pneumonia; Sync: non AAS-related syncope.

Supplementary table 1. Demographic and clinical characteristics of study patients subjected to computed tomography angiography.

**Variable**	**Total patients** (n=162)	**AASs** (n=88)	**AltD** (n=74)	***P*-value**
Female gender	49 (30.2%)	26 (29.5%)	23 (31.1%)	0.83
Age [y]	71 (59-79)	72 (59.5-80)	69 (58-79)	0.51
Hypertension	105 (64.8%)	62 (70.5%)	43 (58.1%)	0.1
Diabetes	12 (7.4%)	7 (8%)	5 (6.8%)	0.77
Dyslipidemia	8 (4.9%)	3 (3.4%)	5 (6.8%)	0.33
Smoking	46 (28.4%)	26 (29.5%)	20 (27%)	0.72
Drug use	2 (1.2%)	2 (2.3%)	0 (0%)	0.19
Coronary art. dis.	17 (10.5%)	6 (6.8%)	11 (14.9%)	0.1
Active cancer	1 (0.6%)	0 (0%)	1 (1.4%)	0.27
Periph. art. dis.	0 (0%)	0 (0%)	0 (0%)	-
Abdominal aortic an.	14 (8.6%)	5 (5.7%)	9 (12.2%)	0.14
Previous AAS	10 (6.2%)	4 (4.5%)	6 (8.1%)	0.35
Systolic BP [mmHg]	140 (120-160)	140 (110-170)	145 (125-160)	0.72
Diastolic BP [mmHg]	80 (70-90)	80 (60-95)	80 (74-90)	0.09
Heart rate [bpm]	74 (67-85)	72 (64-83)	75 (69-90)	0.25
Time from onset [h]	4 (2-9)	3 (2-6)	6 (3-14)	<0.001
WBC count [$*10^3/\mu L$][A]	9.97 (7.68-12.78)	11.66 (9.1-14.35)	8.7 (6.84-10.85)	<0.001
Creatinine [mg/dL][B]	1.01 (0.83-1.2)	1.06 (0.86-1.21)	0.96 (0.82-1.15)	0.11
Troponin T [ng/mL][C]	15 (9-33)	20 (10-41)	11.5 (7.5-19)	0.005
D-dimer [ng/mL][D]	1840 (780-8600)	6301 (1813-28646)	1013.5 (340-1706)	<0.001

[A]n=160; [B]n=159; [C]n=139; [D]n=133. Categorical variables are presented as n (%) and continuous variables as median (25th-75th percentile). AAS= acute aortic syndrome; AltD: alternative diagnoses; an.=aneurysm; art.= artery; BP= blood pressure; dis.= disease; sy.= syndrome; thor.= thoracic.
